# Date palm by-products, fish waste, and *Moringa oleifera* as a cost-effective total mixed ration for fattening lambs: trade-offs between economic benefits and growth performance

**DOI:** 10.3389/fvets.2026.1786930

**Published:** 2026-05-21

**Authors:** Said Al-Khalasi, Abdullah Al-Ghafri, Fahad Al-Yahyaey, Kaadhia Al-Kharousi, Suad Al-Saqri, Zainab Al-Ismaili, Hala Al-Sheibani

**Affiliations:** 1 UNESCO Chair on Aflaj Studies and Socio-hydrology, University of Nizwa, Nizwa, Oman; 2 Animal Production Research Center, Ministry of Agricultural, Fisheries Wealth and Water Resources, Barka, Oman; 3 Animal and Veterinary Sciences, Sultan Qaboos University, Muscat, Oman; 4 Department of Biological Sciences and Chemistry, University of Nizwa, Nizwa, Oman

**Keywords:** alternative feedstuffs, carcass characteristics, date palm by-products, feed digestibility, lamb fattening, *Moringa oleifera*, total mixed ration, economic analysis

## Abstract

**Introduction:**

The cost of animal feed is a serious limiting factor in livestock production in arid and semi-arid regions. This study investigated whether a locally formulated total mixed ration (TMR) based on date palm by-products, fish processing waste, and *Moringa oleifera* could serve as an economical alternative to commercial TMR for fattening Omani lambs.

**Methods:**

Twenty intact male Omani lambs (initial body weight 20.5 ± 2.3 kg, approximately 5 months old) were randomly assigned to two dietary treatments (*n* = 10 per treatment) in a completely randomized design for 84 days. The formulated TMR contained palm fronds (16%), barley grains (20%), fish meal (10%), date syrup (18%), *Moringa oleifera* leaves and stems (34%), and sodium chloride (1%). Productive performance, nutrient digestibility, hematological and biochemical parameters, carcass characteristics, meat quality, and feeding economics were evaluated and compared between the two dietary treatments.

**Results:**

Commercial TMR lambs gained significantly more total weight (11.30 vs. 6.37 kg; *P* < 0.0001) and average daily gain (0.135 vs. 0.076 kg/day) with better feed conversion ratio (6.17 vs. 11.19 kg feed/kg gain; *P* = 0.0011) than lambs fed formulated TMR. Crude protein digestibility was significantly higher in formulated TMR (68.64 vs. 61.35%; *P* = 0.039), whereas neutral detergent fiber digestibility was significantly higher in commercial TMR (49.51 vs. 36.56%; *P* = 0.002). Animals fed commercial TMR had significantly greater slaughter weight (30.86 vs. 25.54 kg; *P* = 0.004), but dressing percentage did not differ between groups. Internal fat deposition was significantly lower (*P* = 0.012) in formulated TMR animals, with 35.4% less total internal fat (1.305 vs. 2.021 kg). Hematological variables were within normal physiological limits in both groups. Blood biochemical analysis showed significant time × treatment interactions for glucose (*P* = 0.001) and calcium (*P* = 0.020). No significant treatment effects were observed for any meat quality attributes, including ultimate pH (5.31 vs. 5.37), cooking loss (27.37 vs. 25.90%), and shear force (3.90 vs. 3.81 kg; all *P* > 0.05). The formulated TMR reduced daily feeding cost by 68.5% and lowered feed cost per kg live weight gain by 41.3% compared to commercial TMR. Hot carcass weight (13.42 ± 2.29 vs. 11.28 ± 2.32 kg; *P* = 0.053) and cold carcass weight (13.07 ± 2.25 vs. 11.03 ± 2.19 kg; *P* = 0.057) were similar between commercial and formulated TMR groups, respectively.

**Discussion/conclusion:**

Despite lower fiber digestibility and metabolizable energy availability than commercial TMR, which translated to reduced growth performance, the formulated TMR offered substantial economic benefits with a 68.5% reduction in daily feeding cost and a 41.3% lower feed cost per kg live weight gain compared to commercial TMR. Meat quality, hot carcass weight, cold carcass weight, and hematological profiles were unaffected, and internal fat deposition was reduced by 35.4%. The formulated TMR represents a viable and cost-effective option for sheep production in arid regions where date palm by-products, fish waste, and *Moringa oleifera* are locally available.

## 1 Introduction

The cost of animal feed is a serious limiting factor in livestock production in arid and semi-arid regions worldwide. The fundamental cause of this is an extreme shortage of freshwater, which severely limits the amount of arable land that can be used to grow feed crops for humans and animals. However, some feeds are locally available, in particular natural rangeland grazing and browsing plants and agricultural by-products, especially those from the date palm, fish-processing waste, and *Moringa spp*. Finding ways to reduce feeding costs through the use of such by-products or alternative feed resources can help promote circular-economy approaches to agriculture and support sustainable livestock production in regions where resources are limited (1, 2).

Sheep production is an integral element of agriculture production systems around the world, contributing to food security and supporting livelihoods in arid and semi-arid environments globally and within Oman (3–5). Feed quality is largely responsible for differences in growth performance, carcass characteristics and profit among animals raised in intensive lamb fattening operations (6, 7). Commercial total mixed rations (TMRs) are common dietary formulations utilized in these settings due to their precise nutritional content and controlled ratios of essential nutrients (8, 9). Yet increasing feed ingredient costs render them economically prohibitive in some cases, especially within countries experiencing water scarcity which precludes most homegrown feed crops (5, 10).

Utilization of locally sourced agricultural by-products and alternative feedstuffs in physiologically appropriate amounts may be one way to decrease production costs while also improving the circular-economy within feed formulations (1, 2). Oman, as well as many other arid-zone countries, produces a variety of feed resources that are underutilized in ruminant diets yet have shown promising nutritive value, including by-products from the date palm (Phoenix dactylifera L.), fish-processing by-products, and *Moringa oleifera* (11–13). Strategically including these ingredients in a complete TMR may be one way to improve the sustainability and affordability of these diets compared to commercial offerings while still achieving acceptable levels of animal performance (4, 14, 15).

Date palm *(Phoenix dactylifera L.)* in arid and semi-arid environments leads to the accumulation of several agricultural residues (11, 14). The global annual production of date palm by-products is estimated to exceed 8 million tons, but they are usually underutilized in animal feeds, which poses a waste management problem (12, 16). Date pits have a crude protein content of 6%−8% and an oil content ranging from 5.77 to 10.71%, with oleic (39.7%−49.7%), lauric (9.7%−24.6%), palmitic (7.8%−14.2%), and linoleic acids (6.2%−17.3%) as the predominant fatty acids, depending on date variety (17). Palm fronds are structural fibers that can be used for rumen filling. However, the lignin content is high in mature palm leaves, which affects fiber digestibility (18, 19). Date palm by-products have been successfully incorporated into the diet of ruminants at different levels, and their nutritive value has been improved through protein supplementation or processing techniques to enhance digestibility (18, 20).

*Moringa oleifera Lam*. is a multipurpose fast-growing tree that was domesticated in India but has since been widely cultivated in tropical and subtropical regions around the world (21, 22). Moringa leaves are high in crude protein (25%−30%) and have a favorable amino acid profile with a high content of essential amino acids, such as lysine, methionine, and tryptophan (23). In addition to their nutritional value, Moringa leaves also contain bioactive phytochemicals such as phenolic acids, flavonoids, and glucosinolates, which have antioxidant, antimicrobial, and anti-inflammatory properties (24, 25). Several studies have found that supplementation with moringa leaves can improve growth performance, enhance immune function, and reduce oxidative stress in livestock (26, 27).

Dietary fiber is an important aspect of animal feed quality and affects dry matter intake, nutrient digestibility, and metabolism in ruminants (28, 29). Fiber is quantitatively described by its content (neutral detergent fiber (NDF) and acid detergent fiber (ADF)) as well as its qualitative aspect (cellulose, hemicellulose, and lignin) (30, 31). Fiber digestibility is generally low because of the high lignin content, and high levels of fiber in the diet may compromise nutrient digestibility and reduce energy density (30, 31). Although fiber is necessary for normal rumen function, excess fiber fills the rumen and reduces dry matter intake. There is no single ideal level of dietary NDF for finishing lambs because both the production system and fiber source affect the recommendation. Dietary NDF levels of 25%−35% of dry matter have been recommended for intensive finishing systems to maintain rumen health without sacrificing energy intake (32, 33). However, several authors have argued that NDF levels as low as 20%−25% of dry matter is adequate if highly digestible fiber sources are fed in high concentrate diets (34, 35). Regardless of the recommended level, fiber quality will ultimately dictate animal performance because increased fiber digestibility will allow for increased intake and improved feed efficiency (36).

Besides sources of roughage, locally available protein ingredients should also be considered to economically formulate diets for intensive lamb fattening. Fish by-products and waste products, including fish meal and silage, represent potential sources of high-quality protein for animal feed (13, 37). Their protein is of high biological value, with a favorable amino acid profile rich in essential amino acids such as lysine and methionine (15). Fish meal also has high rumen undegradability, which may improve amino acid availability for intestinal digestion and absorption (38). Fish by-products also contain omega-3 polyunsaturated fatty acids, which have been found to have positive effects on meat quality (38).

The Omani sheep is a fat-tailed breed adapted to harsh arid environments, characterized by moderate heat stress tolerance and low availability of high-quality feed (4, 15). Omani sheep are among the most important local breeds and play a significant role in sheep production and fattening in the Sultanate of Oman. Optimizing feeding for fattening is thus of great importance for local sheep production and the sustainable use of this animal genetic resource (4, 15).

This study compared a formulated TMR using date palm by-products, fish waste, and *Moringa oleifera* with a commercial TMR for growth performance, physiological parameters, carcass characteristics, and meat quality in fattening Omani lambs. It was hypothesized that the formulated TMR would provide adequate nutritional value for acceptable performance while offering economic advantages over the commercial TMR.

## 2 Materials and methods

### 2.1 Animals, housing, and experimental design

The feeding experiment was conducted at the Animal Production Center (APC), Al-Rumais, Wilayat Barka, Sultanate of Oman (latitude, approximately 23.68° N; longitude, approximately 57.99° E). The experimental period lasted from April 21, 2025, to September 14, 2025. During the experimental period, the ambient temperature at the experimental farm was 28–35 °C. 20 intact male Omani lambs (initial body weight 20.5 ± 2.3 kg, approximately 5 months old) were assigned randomly to two dietary treatments (*n* = 10 per treatment) in a completely randomized design. Animals were housed in individual pens under a shaded roofed structure with concrete floors (1.5 × 2.0 m) with *ad libitum* access to fresh water. After a 14-day adaptation period, an 84-day experimental period was followed.

### 2.2 Dietary treatments and feeding management

The formulated TMR contained palm fronds (16%), barley grains (20%), fish meal (10%), date syrup (18%), *Moringa oleifera* leaves and stems (34%), and sodium chloride (NaCl, non-iodized) (1%). Palm fronds were chopped to approximately 2–3 cm pieces and sun-dried. *Moringa oleifera* leaves and stems were harvested after approximately 60 d of growth. The commercial TMR was a commercially available pelleted lamb fattening ration, which formulated TMR balanced in accordance with the NRC guidelines (39). Experimental diets were offered once per day (08:30 h) at 3.5% of the mean body weight according to NRC (2007) recommendations. The feed offered and refused was weighed daily to determine individual animal feed intake.

### 2.3 Chemical analysis

Feed samples were analyzed for dry matter (DM) using AOAC method 934.01, crude protein (CP) by the Kjeldahl method (AOAC method 976.05), ether extract (EE) by Soxhlet extraction (AOAC method 920.39), and ash content by combustion in a muffle furnace at 550 °C for 4 h (AOAC method 942.05), all according to AOAC (2005) procedures (40). Neutral detergent fiber (NDF) and acid detergent fiber (ADF) were analyzed using heat-stable amylase and expressed exclusive of residual ash, as described by Van Soest et al. (41). Calcium (Ca) and phosphorus (P) concentrations were determined using atomic absorption spectrophotometry following acid digestion (AOAC method 968.08 for calcium; AOAC method 965.17 for phosphorus) (40). Metabolizable energy (ME) content was calculated using prediction equations (39).

### 2.4 Digestibility trial

Digestibility was determined in a 7-day trial (days 56–63) using four animals per treatment group. Complete fecal and urine output was recorded over seven consecutive days following adaptation to collection for 3 days. Fecal and urine samples were separated utilizing a collection device placed beneath each cage. Samples were passed through a collector with small holes that caught feces and allowed urine to drip through into plastic containers below. This method prevented feces from being placed into urine and urine from being placed into feces. Fresh fecal mass was determined daily, and a fresh 10% subsample of feces was collected each day and stored at −20 °C. Urine volume was measured each day and was preserved with 20 ml of 10 M H_2_SO_4_ to prevent volatilization of nitrogen. Liquid paraffin was sprayed on top of urine sample each day to a final volume of 10 mL to prevent nitrogen loss via evaporation. Samples were composited per animal at the end of the seven-day collection period and then sub sampled for analysis. Urine samples were stored at 4 °C during collection and then sub sampled for analysis. Apparent digestibility coefficients (DC) of DM, CP, NDF, ADF, and EE were calculated using the formula:(Intake – Fecal Output)/Intake × 100%.

### 2.5 Growth performance

Body weight was recorded twice weekly, following an overnight fasting period. Average daily gain (ADG), total weight gain, feed efficiency (FE), and feed conversion ratio (FCR) were calculated.

### 2.6 Blood sampling and analysis

Blood samples were obtained by jugular venipuncture at day 0 (before implantation) and day 84 (end of experiment) into vacutainer tubes. Blood samples for hematological variables were collected into EDTA tubes (3 mg/ml EDTA) and analyzed using an automated hematology analyzer within 2 h of collection (Sysmex XN-1,000, Sysmex Corporation, Kobe, Japan). Intra-assay CV% were less than 3% for red blood cell count, hemoglobin and hematocrit, and less than 5% for white blood cell differential counts using this analyzer, per manufacturer specifications and previously reported validation (42). Blood samples for biochemical variables were collected into plain vacutainer tubes and allowed to clot at room temperature for 30 min prior to centrifugation at 3,000 rpm for 15 min. Serum was then separated and glucose, total protein, albumin, blood urea nitrogen, creatinine, calcium and phosphorus concentrations were determined using commercial kits on an automated biochemistry analyzer (Mindray BS-400, Mindray Medical International, Shenzhen, China). All assays were conducted per manufacturer instructions, with intra-assay CV% below 5% and inter-assay CV% below 8% for all parameters previously reported for validation of ovine serum biochemistry (32, 43).

### 2.7 Slaughter and carcass evaluation

Animals were slaughtered following Islamic halal procedures following overnight fasting. Animals were weighed before slaughter (pre-slaughter weight). Hot and cold carcass weights (following 24-h chilling at 4 °C) were recorded, and the dressing percentage was calculated. The internal fat depots, livers, kidneys, pelvis, and heart were dissected and weighed separately.

### 2.8 Meat quality assessment

Longissimus dorsi samples collected at the 12th rib were analyzed for meat quality. Meat quality parameters included ultimate pH, cooking loss, Warner-Bratzler shear force, water-holding capacity, instrumental color (L^*^, a^*^, b^*^), and sarcomere length. Standard laboratory methods were used for all evaluations using calibrated instruments under constant temperature and processing conditions to ensure the repeatability and accuracy of the measurements (44). All animal experiments were approved by the Animal Ethics and Professional Conduct Committee under the Ministry of Agriculture Fisheries Wealth and Water Resources, Sultanate of Oman (Ethical approval protocol #: MAFWR-AHPC-AEC-2025-047), and were performed according to institutional guidelines.

### 2.9 Economic analysis

The economic evaluation of the feeding system was based on ingredient costs, daily feeding cost, cost/kg of live weight gain, and cost/kg of cold carcasses.

### 2.10 Statistical analysis

Data was analyzed using the IBM Statistical Package for the Social Sciences software program (version 27.0; IBM Corporation, Armonk, NY, USA). Weekly feed intake and biweekly body weight were analyzed as repeated measures using Linear Mixed Model (LMM) procedure with treatment (commercial TMR vs. formulated TMR) and week as fixed effects, with animal (nested within treatment) as random effect to consider repeated measures within animal and using time as repeated measures. An unstructured covariance matrix and Greenhouse-Geisser adjustment were used to estimate the covariance parameters of repeated measures and when sphericity was violated, respectively. Independent samples *t*-test was conducted to determine differences between treatment groups for carcass characteristics, meat quality parameters, blood hematological parameters and serum biochemical parameters that were collected at only one time point. Blood biochemical and hematological parameters collected at two time points (day 0 and day 84) were analyzed using mixed model repeated measures procedures with treatment, time as fixed effects, treatment × time interaction as fixed effect and animal as random effect. Data are reported as mean ± SEM. *P* < 0.05 was considered statistically significant.

## 3 Results

### 3.1 The chemical composition of feed ingredients

The mean values for the chemical compositions of the ingredients formulated in the TMR are presented in Table 1. The percentage composition of ingredients included in the TMR formulation varied, with *Moringa oleifera* accounting for 35%, barley 20%, date syrup 18%, date palm fronds 16%, and waste fish meal 10% of the diet. Sodium chloride (NaCl, non-iodized) was supplemented with formulated TMR at a rate of 1%.

**Table 1:** Chemical composition of feed ingredients used in formulated total mixed ration (% dry matter basis unless otherwise stated).

Component	*Moringa oleifera*	Barley	Date syrup	Date palm fronds	Fish meal	Sodium chloride (NaCl, non-iodized)
Dry matter (%)	93.03	89.5	56.0	91.2	92.81	96.0
Crude protein	20.85	10.5	1.2	4.2	42.5	0
Ether extract	4.15	2.1	0	4.78	15.25	0
Ash	14.35	2.6	3.5	12.8	28.52	100
NDF	48.0	20.0	0	48.6	23.0	0
ADF	35.2	5.8	0	39.8	3.6	0
Hemicellulose	12.8	14.2	0	8.8	19.4	0
Cellulose	28.5	4.9	0	31.2	2.8	0
Lignin	6.7	0.9	0	8.6	0.8	0
NFE	12.65	64.8	95.3	29.62	10.73	0
Gross energy (kJ/g)	140.37	129.25	165.4	198.28	139.49	0
Calcium	2.427	0.05	0.02	0.65	5.36	0
Phosphorus	0.244	0.38	0.01	0.08	3.36	0
Ca:P ratio	9.95:1	0.13:1	2:1	8.13:1	1.60:1	-

Reported NDF and ADF values for fish meal represent detergent insoluble non-carbohydrate material (connective tissue proteins, collagen, and bone matrix) that are intrinsic to the Van Soest detergent fiber procedure when applied to ingredients of animal origin (94), not structural plant fiber. Cellulose and hemicullose values are estimates calculated as ADF minus lignin, and NDF minus ADF, respectively, and are therefore subject to the same interference.

Dry matter values ranged from 96% in sodium chloride (NaCl, non-iodized) to 93.03 and 92.81% in *Moringa oleifera* and waste fish meal, respectively, while it was 56% in date syrup. The protein content of ingredients included in the TMR formulation was different, varying from 1.2% in date syrup to 42.5% in waste fish meal and 20.85% in Moringa oleifera. The ether extract content of the ingredients was higher in waste fish meal (15.25%) and lower in date syrup (0%), followed by *Moringa oleifera* (4.15%) and date palm fronds (4.78%).

The fiber fractions also varied widely between ingredients, with neutral detergent fiber being high in *Moringa oleifera* and date palm fronds (48 and 48.6%, respectively), while it was 23 and 20% in waste fish meal and barley, respectively. Acid detergent fiber content ranged from 3.6% in waste fish meal to 39.8% in date palm fronds. The hemicellulose content of the ingredients ranged from 8.8% in date palm fronds to 19.4% in waste fish meal. Ash values were high in waste fish meal (28.52%) and *Moringa oleifera* (14.35%), while the lowest values were in barley (2.6%). Gross energy (GE) values in ingredients ranged from 129.25 kJ/g in barley to 198.28 kJ/g in date palm fronds. *Moringa oleifera* and waste fish meal had intermediate values of GE (140.37 and 139.49 kJ/g, respectively). The percentage values of calcium and phosphorus were highest in waste fish meal (5.36 and 3.36%, respectively), followed by *Moringa oleifera* (2.427 and 0.244%, respectively).

### 3.2 The chemical composition of the commercial and formulated total mixed rations

The chemical compositions of commercial and formulated TMRs are listed in Table 2. The dry matter content was significantly higher (95.02%) in the commercial TMR than in the formulated TMR (93.32%). Crude protein levels were similar (13.12 and 13.55%, respectively) between TMRs. The ether extract value was slightly lower in the formulated TMR (3.91%) than that in the commercial TMR (4.41%).

**Table 2:** Chemical composition and nutritional characteristics of commercial and formulated total mixed rations (% dry matter basis unless otherwise stated).

Component	Commercial TMR	Formulated TMR
Dry matter (%)	95.02	93.32
Crude protein	13.12	13.55
Ether extract	4.41	3.91
Ash	11.96	11.64
NDF	38.25	33.22
ADF	20.66	24.97
Hemicellulose	17.59	8.25
Cellulose	16.8	20.1
Lignin	3.86	4.87
NFE	51.45	48.18
TDN	72.81	69.45
Gross energy (MJ/kg DM)	17.39	17.47
ME (MJ/kg DM)	10.98	10.48
Calcium	1.12	1.24
Phosphorus	0.68	0.52
Ca:P ratio	1.65:1	2.38:1
Diet formulation	Commercial pellets	Moringa 35%, Barley 20%, Date syrup 18%, Palm fronds 16%, Fish meal 10%, Sodium chloride (NaCl, non-iodized) 1%

The neutral detergent fiber content was higher (38.25%) in the commercial TMR than in the formulated TMR (33.22%). The acid detergent fiber contents were 24.97 and 20.66% in the formulated TMR and commercial TMR, respectively. The hemicellulose fraction was higher (17.59%) in the commercial TMR than that in the formulated TMR (8.25%). Ash values were non-significantly different (11.96 and 11.64% in the commercial and formulated TMR, respectively). Energy values showed that the total digestible nutrients were 72.81 and 69.45% in the commercial TMR and formulated TMR, respectively, whereas the nitrogen-free extract was 51.45 and 48.18% in the commercial TMR and formulated TMR, respectively. The GE was almost equal in the commercial and formulated TMR (17.39 and 17.47, respectively). The metabolizable energy (ME) was higher in the commercial TMR (10.98 MJ/kg DM) than in the formulated TMR (10.48 MJ/kg DM).

### 3.3 Apparent nutrient digestibility

**Figure 1:**
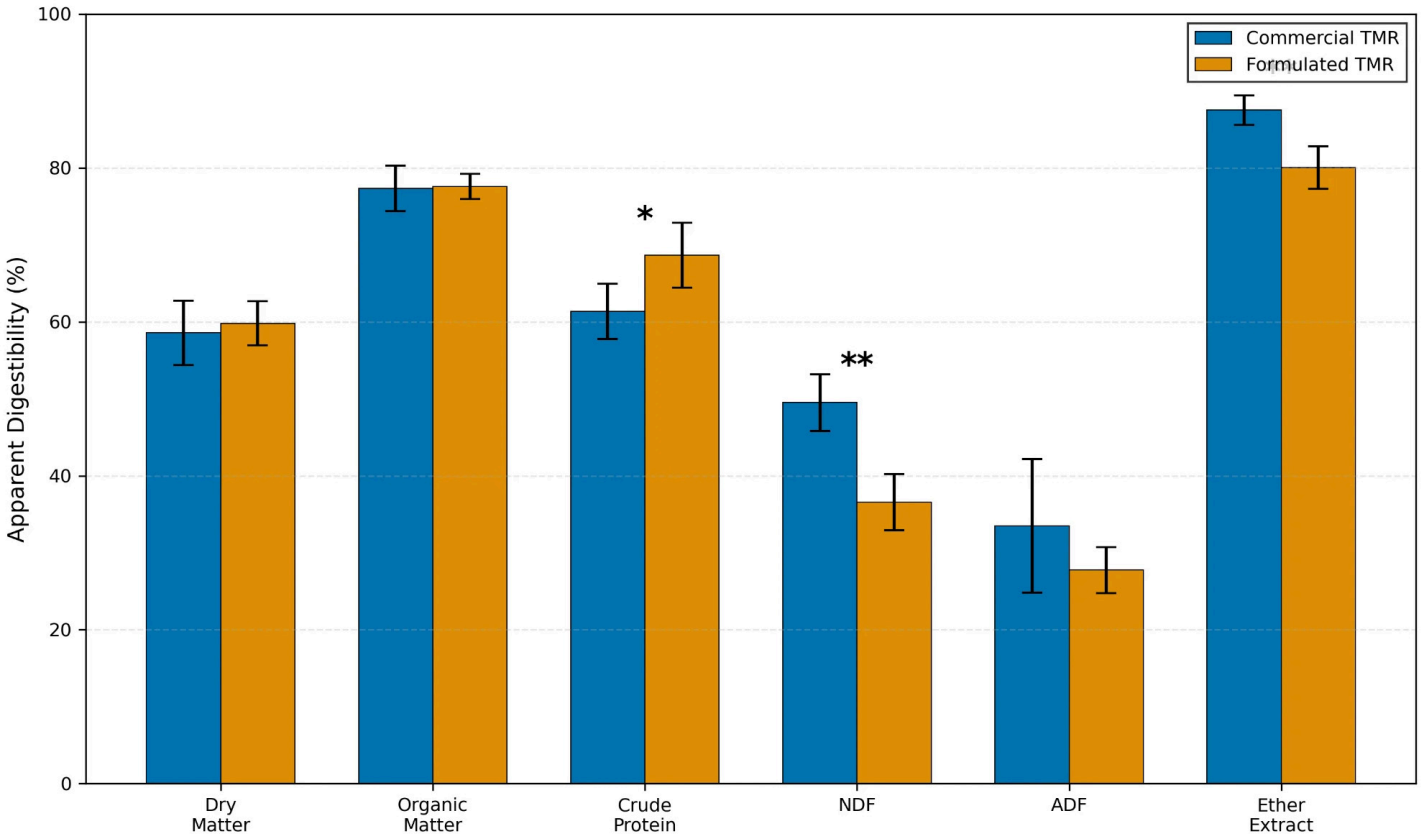
Bar chart comparing apparent digestibility percentages of dry matter, organic matter, crude protein, NDF, ADF, and ether extract between lambs fed commercial TMR (blue bars) and locally formulated TMR (orange bars). Significant differences indicated for crude protein (^*^P < 0.05) and NDF (^**^P < 0.01). Error bars = SEM; *n* = 4 per group.

Nutrient digestibility of the commercial and formulated TMRs is presented in Table 3 and Figure 1. The apparent digestibility of DM was not different (58.57 ± 4.20% and 59.82 ± 2.87%) between the two treatments (*P* = 0.641). Similarly, organic matter digestibility was not significantly different between commercial and formulated TMRs (77.36 ± 2.96 and 77.60 ± 1.64%, respectively; *P* = 0.891).

**Table 3:** Apparent digestibility coefficients (%) of major nutrients in commercial and formulated total mixed rations.

Nutrient	Commercial TMR (%)	Formulated TMR (%)	*p*-value
Dry matter (DM)	58.57 ± 4.20	59.82 ± 2.87	0.641
Organic matter (OM)	77.36 ± 2.96	77.60 ± 1.64	0.891
Crude protein (CP)	61.35 ± 3.59	68.64 ± 4.21	0.039^*^
Neutral detergent fiber (NDF)	49.51 ± 3.69	36.56 ± 3.64	0.002^**^
Acid detergent fiber (ADF)	33.48 ± 8.68	27.74 ± 2.98	0.257
Ether extract (EE)	87.52 ± 1.92	80.06 ± 2.77	0.004^**^

Values are means ± SEM (*n* = 4 per treatment). ^*^P < 0.05; ^**^P < 0.01. NDF, neutral detergent fiber; ADF, acid detergent fiber.

CP digestibility was significantly higher in the formulated TMR (68.64 ± 4.21%) than in the commercial TMR (61.35 ± 3.59%; *p* = 0.039), which showed an 11.9% improvement in formulated TMR. In contrast, NDF digestibility was significantly higher (49.51 ± 3.69%) in commercial TMR than in formulated TMR (36.56 ± 3.64%; *p* = 0.002), which indicated 26.2% decrease in the formulated TMR. Although ADF digestibility tended to be lower in formulated TMR (27.74 ± 2.98%) than in commercial TMR (33.48 ± 8.68%), it was not significant (*p* = 0.257) because of the high standard deviation in the control group. EE digestibility was significantly higher in commercial TMR (87.52 ± 1.92%) than in formulated TMR (80.06 ± 2.77%; *p* = 0.004) (Figure 1).

### 3.4 Feed intake and conversion efficiency

Feeding behavior, performance, and efficiency of the two groups are shown in Table 4. Total weight gain and average daily gain were significantly higher in the commercial TMR (11.30 ± 1.18 kg and 0.135 ± 0.014 kg/d, respectively) than in the formulated TMR group (6.37 ± 1.87 kg and 0.076 ± 0.022 kg/d, respectively) (*t* = 7.05, *p* < 0.0001). Although the total feed intake was not different (69.29 ± 4.14 and 66.16 ± 8.33 kg, respectively; *p* = 0.306), feed efficiency ratio (FER) was significantly higher in the commercial TMR group (0.163 ± 0.014 kg gain/kg feed) than in the formulated TMR group (0.095 ± 0.021 kg gain/kg feed; *p* < 0.0001). In contrast, feed conversion ratio (FCR) was significantly lower in the commercial TMR group (6.17 ± 0.50 kg feed/kg gain) than in the formulated TMR group (11.19 ± 3.40 kg feed/kg gain; *p* = 0.0011).

**Table 4:** Growth performance and feed efficiency of lambs fed commercial and formulated total mixed rations over 84 days.

Parameter	Commercial TMR	Formulated TMR	*p*-value
Initial BW (kg)	19.56 ± 3.09	19.17 ± 2.79	0.314
Final BW (kg)	30.86 ± 3.25	25.54 ± 3.90	0.001^**^
Total weight gain (kg)	11.30 ± 1.18	6.37 ± 1.87	<0.0001
Average daily gain (kg/d)	0.135 ± 0.014	0.076 ± 0.022	<0.0001^***^
Total feed intake (kg)	69.29 ± 4.14	66.16 ± 8.33	0.306
FER (kg gain/kg feed)	0.163 ± 0.014	0.095 ± 0.021	<0.0001^***^
FCR (kg feed/kg gain)	6.17 ± 0.50	11.19 ± 3.40	0.0011^**^

Values are means ± SEM (*n* = 10 per treatment). ^**^P < 0.01; ^***^P < 0.001. BW, body weight; ADG, average daily gain; FER, feed efficiency ratio; FCR, feed conversion ratio.

Repeated-measures ANOVA was performed to compare feed intake at weeks 0–12. It revealed a highly significant main effect of weekly feed intake (*F* = 6.46, *P* < 0.001) and showed that the feed intake of lambs in both groups changed significantly during the 12 weeks. The between-subject analysis showed that the overall mean feed intake was not significantly different between the commercial and formulated TMR groups (*F* = 1.73, *p* = 0.205). The initial multivariate tests indicated the potential existence of a week × group interaction (Pillai's Trace, *F* = 3.75, *p* = 0.036); however, after the correction for sphericity using Greenhouse-Geisser adjustment, the interaction effect became non-significant (*F* = 1.71, *p* = 0.174), suggesting that both groups followed a similar intake pattern over 12 weeks.

### 3.5 Body weight changes

**Figure 2:**
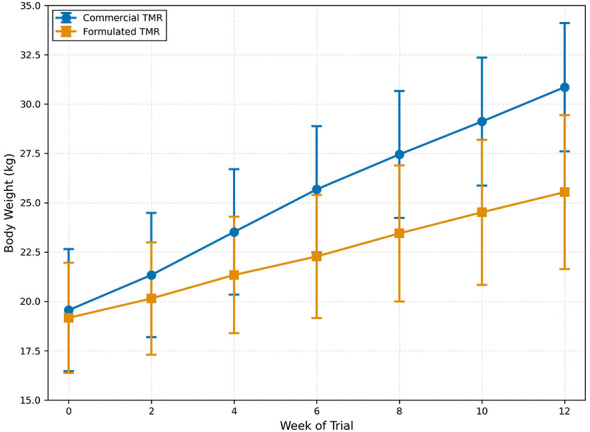
Body weight (kg) of lambs fed commercial (blue circles) or locally formulated (orange squares) total mixed ration (TMR), recorded every 2 weeks throughout a 12 wks feeding trial. All lambs gained weight linearly throughout the trial and lambs fed commercial TMR attained greater BW from wk 0 to wk 12. Mean ± SD; *n* = 10/group.

Weight measurements of the animals from the two treatments are shown in Figure 2. Repeated-measures ANOVA revealed a highly significant main effect of time (*F* = 152.33, *p* < 0.001, Partial η^2^ = 0.894) and showed that the body weight of lambs in both groups increased significantly over the experimental weeks. A significant main effect of group was detected (*F* = 4.713, *p* = 0.044, Partial η^2^ = 0.208), with the commercial TMR group exhibiting consistently higher mean body weight (25.46 ± 1.06 kg) than the formulated TMR group (22.20 ± 1.06 kg). A significant time × group interaction was observed (*F* = 9.401, *p* < 0.001, Partial η^2^ = 0.343), demonstrating that the growth trajectory of the lambs differed between the two groups. Although both groups showed weight gain over time, the commercial TMR group exhibited a steeper and more sustained increase than the formulated TMR group, with the gap between groups widening in the later weeks of the trial. Initial body weights in both groups were comparable (19.56 ± 3.09 and 19.17 ± 2.79 kg, respectively), but by week 12, the commercial TMR group reached 30.86 ± 3.25 kg, while the formulated TMR group achieved only 25.54 ± 3.90 kg, representing a 17.2% difference in final body weight.

### 3.6 Hematological and biochemical parameters

The hematological responses of the animals fed commercial and formulated TMRs are presented in Table 5. Animals from the two treatments did not differ (*p* > 0.05) in any hematological parameters. There was a significant effect of time (*p* < 0.05) on a number of variables (red blood cell count, hemoglobin, hematocrit, mean corpuscular hemoglobin, red cell distribution width, monocytes, basophils, and reticulocyte hemoglobin content), which changed significantly across the time points regardless of the group. The values for all parameters were within the reference ranges for healthy sheep.

**Table 5:** Hematological parameters of Omani lambs fed commercial or formulated TMR.

Parameter	Unit	Commercial TMR pre	Commercial TMR post	Formulated TMR pre	Formulated TMR post	SEM	*P*-value treatment	*P*-value time	*P*-value *T* × *T*	Reference range
RBC	×10^12^/L	11.20	12.40	11.50	12.60	0.35	0.654	0.021^*^	0.892	9.0–15.0
Hemoglobin	g/dl	10.80	11.90	10.50	11.60	0.28	0.523	0.003^**^	0.871	9.0–15.0
Hematocrit	%	32.50	35.80	31.90	35.20	0.92	0.612	0.001^**^	0.921	27.0–45.0
MCV	Fl	29.20	28.80	28.60	28.90	0.45	0.782	0.652	0.445	28.0–40.0
MCH	Pg	9.70	9.40	9.20	9.50	0.18	0.892	0.038^*^	0.125	8.0–12.0
MCHC	g/dl	33.20	33.10	32.90	33.40	0.35	0.721	0.523	0.287	31.0–34.0
RDW	%	19.80	18.20	20.10	18.50	0.52	0.634	0.012^*^	0.923	16.0-25.0
WBC	×10^9^/L	7.20	7.80	7.50	8.10	0.42	0.512	0.218	0.891	4.0–12.0
Neutrophils	×10^9^/L	2.80	3.10	2.90	3.20	0.25	0.723	0.142	0.956	0.7–6.0
Lymphocytes	×10^9^/L	3.80	4.10	4.00	4.30	0.32	0.623	0.234	0.891	2.0–9.0
Monocytes	×10^9^/L	0.40	0.40	0.40	0.40	0.08	0.891	0.623	0.812	0.0–0.75
Eosinophils	×10^9^/L	0.20	0.20	0.20	0.20	0.05	0.956	0.812	0.923	0.0–1.0
Basophils	×10^9^/L	0.00	0.00	0.00	0.00	0.02	0.891	0.891	0.956	0.0–0.3
Platelets	×10^9^/L	442.00	485.00	458.00	502.00	28.50	0.634	0.089	0.812	200–800

Values are means (*n* = 10 per treatment group). ^*^P < 0.05; ^**^P < 0.01. RBC, red blood cell count; MCV, mean corpuscular volume; MCH, mean corpuscular hemoglobin; MCHC, mean corpuscular hemoglobin concentration; RDW, red cell distribution width; WBC, white blood cell count; SEM, standard error of mean; T × T, time × treatment interaction. Reference ranges represent normal values for healthy sheep.

The serum biochemical parameters of animals fed commercial and formulated TMRs are presented in Table 6. The multivariate test indicated a highly significant overall effect of time (Pillai's trace = 0.889, *p* < 0.001) and a significant time × treatment interaction (Pillai's trace = 0.740, *p* = 0.020). The albumin concentration decreased significantly over time (*F* = 9.203, *p* = 0.007), with no differences between treatments (*p* = 0.855). There was a strong time effect for glucose (*F* = 60.172, *p* < 0.001), with glucose levels dropping sharply from pre- to post-treatment. A significant time × treatment interaction (*F* = 14.919, *p* = 0.001) indicated that the pattern of reduction differed between the groups, with a more moderate decrease in the formulated TMR group compared with the pronounced decline in the control group.

**Table 6:** Serum biochemical parameters of Omani lambs fed commercial or formulated TMR.

Parameter	Unit	Commercial TMR pre	Commercial TMR post	Formulated TMR pre	Formulated TMR post	SEM	*P*-value treatment	*P*-value time	*P*-value *T* × *T*
Total protein	g/L	68.50	66.20	67.80	65.90	1.82	0.712	0.089	0.891
Albumin	g/L	32.80	29.50	33.20	30.10	1.15	0.855	0.007^**^	0.623
Globulin	g/L	35.70	36.70	34.60	35.80	1.45	0.523	0.234	0.756
A/G ratio	—	0.92	0.80	0.96	0.84	0.06	0.634	0.067	0.892
Glucose	mmol/L	4.85	3.12	4.78	3.68	0.18	0.089	<0.001^***^	0.001^**^
Urea	mmol/L	6.20	6.80	6.50	7.10	0.35	0.423	0.123	0.712
Creatinine	μmol/L	88.50	92.30	86.20	91.80	3.25	0.567	0.156	0.834
Calcium	mmol/L	2.45	2.32	2.38	2.48	0.08	0.456	0.910	0.020^*^
Phosphorus	mmol/L	2.18	2.25	2.15	2.22	0.12	0.712	0.234	0.891
Magnesium	mmol/L	1.15	1.22	1.18	1.24	0.06	0.623	0.145	0.923
Sodium	mmol/L	145.20	146.80	144.80	146.20	2.15	0.734	0.089	0.812
Potassium	mmol/L	4.80	5.10	4.90	5.20	0.18	0.567	0.067	0.756
Chloride	mmol/L	105.80	107.20	106.20	107.80	1.85	0.623	0.123	0.891
Copper	μmol/L	18.50	19.20	15.80	16.40	0.85	0.018^*^	0.234	0.712
Iron	μmol/L	28.60	30.20	24.50	25.80	1.25	0.005^**^	0.145	0.567
Zinc	μmol/L	14.20	15.10	13.80	14.60	0.65	0.523	0.089	0.834
AST	U/L	85.20	92.80	82.60	89.50	3.85	0.412	0.067	0.923
ALT	U/L	28.50	31.20	27.80	30.50	1.45	0.634	0.123	0.812
ALP	U/L	185.60	198.20	182.40	195.80	6.25	0.567	0.089	0.756
CK	U/L	142.50	198.60	148.20	225.80	8.95	0.031^*^	0.005^**^	0.234

A/G, albumin/globulin ratio; AST, aspartate aminotransferase; ALT, alanine aminotransferase; ALP, alkaline phosphatase; CK, creatine kinase; SEM, standard error of mean; T × T, time × treatment interaction. ^*^P < 0.05; ^**^P < 0.01; ^***^P < 0.001. Values are means (*n* = 10 per treatment group). Multivariate test results: time effect (Pillai's trace = 0.889, *P* < 0.001); Time × Treatment interaction (Pillai's trace = 0.740, *P* = 0.020).

Calcium showed no time effect (*p* = 0.910), but the time × treatment interaction was significant (*F* = 6.492, *p* = 0.020), indicating contrasting calcium dynamics between the groups; calcium levels declined in the commercial TMR group but increased slightly in the formulated TMR group. Copper levels were significantly affected by treatment (*F* = 6.736, *P* = 0.018), with the formulated TMR group showing lower copper concentrations. Iron levels were also significantly affected by the treatment (*F* = 10.295, *p* = 0.005), with the commercial TMR group showing higher iron levels. Creatine kinase (CK) concentration increased significantly over time (*F* = 10.324, *p* = 0.005) and was significantly higher in the formulated TMR group (*F* = 5.487, *p* = 0.031).

### 3.7 Slaughter characteristics and carcass yield

**Figure 3:**
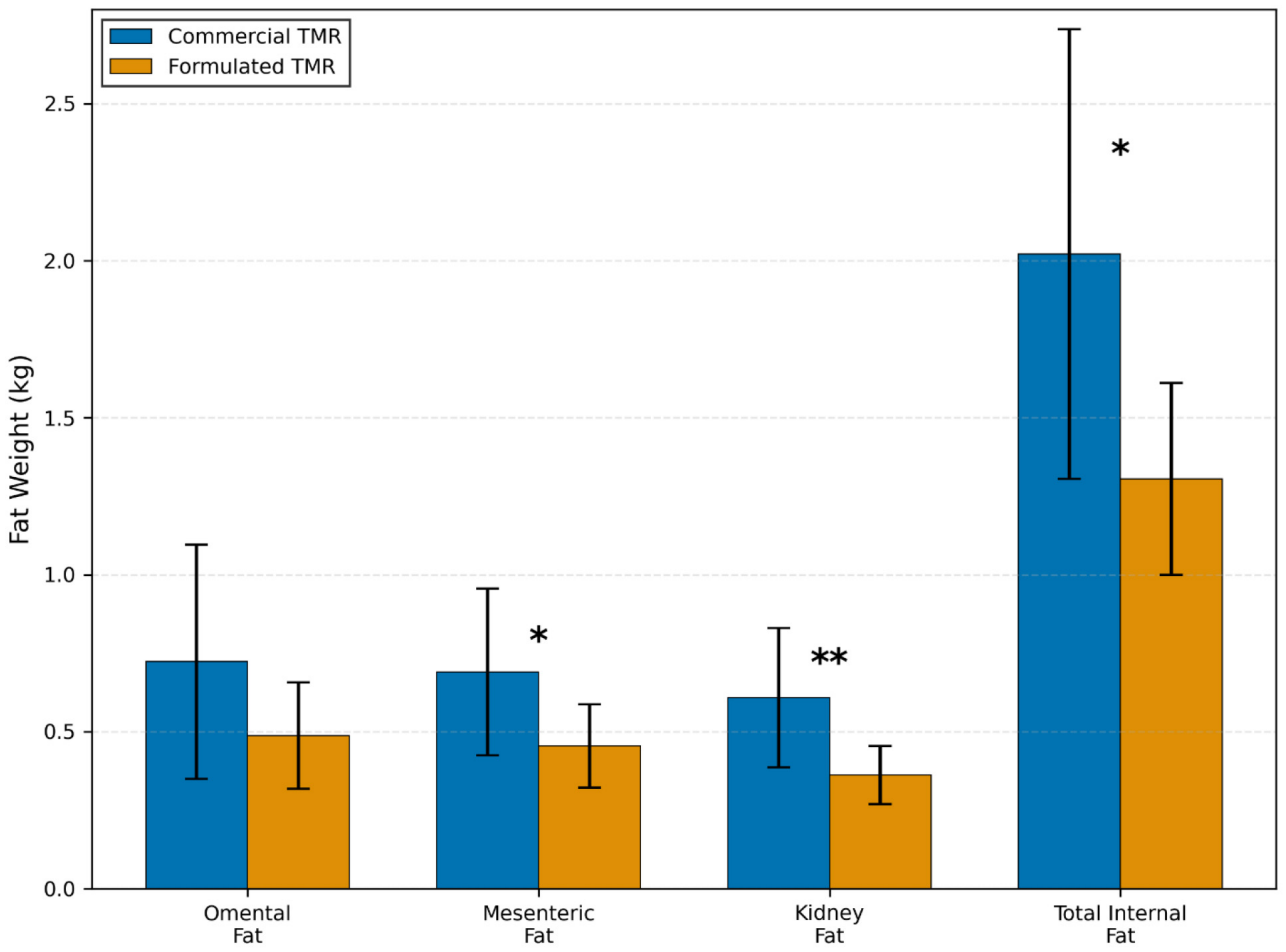
Weight (kg) of individual internal fat depots (omentum, fat covering the stomach; mesenteric, fat along the intestine lining; kidney, perirenal fat), as well as their combined total as whole internal fat, for lambs fed either a commercial or locally formulated TMR. Locally Formulated TMR decreased mesenteric fat deposition (*P < 0.05), kidney fat deposition (**P < 0.01), and total internal fat deposition (*P < 0.05). There was no significant difference between groups for omental fat. Mean ± SEM; *n* = 10 per group.

As are illustrated in Table 7, slaughter weights were significantly higher in animals fed the commercial TMR compared to the formulated TMR (30.86 ± 3.25 and 25.54 ± 3.91 kg, respectively; *P* = 0.004). The empty body weight was also significantly higher in animals fed the commercial TMR than the formulated TMR (28.57 ± 3.18 and 22.73 ± 4.08 kg, respectively; *P* = 0.002). Hot carcass weight tended to be higher in the commercial TMR group (13.42 ± 2.29 kg) than in the formulated TMR group (11.28 ± 2.32 kg), although the difference was not statistically significant (*P* = 0.053). The cold carcass weight followed a similar pattern (13.07 ± 2.25 and 11.03 ± 2.19 kg for the commercial and formulated TMR groups, respectively; *P* = 0.057). Carcass shrinkage did not differ between the two treatments (2.63 ± 1.47 and 2.07 ± 1.21% for the commercial and formulated TMR groups, respectively; *P* = 0.582). The dressing out percentage was not different between the two dietary treatments (43.23 ± 3.86 and 44.43 ± 7.95% for the commercial and formulated TMR groups, respectively; *P* = 0.709). As shown in Figure 3, omental fat weight tended to be higher in commercial TMR animals than in formulated TMR animals (0.723 ± 0.373 and 0.488 ± 0.169 kg, respectively; *P* = 0.086), indicating a 32.5% reduction. Mesenteric fat deposition was significantly lower in formulated TMR animals (0.455 ± 0.133 and 0.690 ± 0.266 kg for the formulated and commercial TMR groups, respectively; *P* = 0.023), indicating a 34.1% reduction. Kidney fat showed the most pronounced difference between groups, with formulated TMR animals depositing significantly less perirenal adipose tissue (0.362 ± 0.093 and 0.608 ± 0.222 kg for the formulated and commercial TMR groups, respectively; *P* = 0.005), indicating a 40.5% reduction. Total internal fat deposition was significantly lower in formulated TMR animals (1.305 ± 0.305 and 2.021 ± 0.716 kg for the formulated and commercial TMR groups, respectively; *P*= 0.012), indicating a 35.4% reduction.

**Table 7:** Slaughter characteristics and carcass measurements of lambs fed commercial and formulated total mixed rations.

Parameter	Commercial TMR	Formulated TMR	*P*-value
Slaughter weight (kg)	30.86 ± 3.25	25.54 ± 3.90	0.005^**^
Empty body weight (kg)	28.57 ± 3.18	22.73 ± 4.08	0.002^**^
Hot carcass weight (kg)	13.42 ± 2.29	11.28 ± 2.32	0.053
Cold carcass weight (kg)	13.01 ± 2.24	10.99 ± 2.31	0.063
Carcass shrinkage (%)	2.63 ± 1.47	2.07 ± 1.21	0.364
Dressing percentage (%)	43.06 ± 2.59	43.81 ± 3.13	0.598
Liver (kg)	0.47 ± 0.09	0.48 ± 0.08	0.996
Heart (kg)	0.11 ± 0.02	0.11 ± 0.03	0.757

Values are means ± SEM (*n* = 10 per treatment). ^**^P < 0.01.

The non-carcass components (head, skin, wool, lung, trachea, heart, liver, and kidney) showed no significant differences between the two groups. Head weight (1.09 ± 0.12 and 1.09 ± 0.13 kg for the commercial and formulated TMR groups, respectively; *p* = 0.922), skin and wool weights (3.62 ± 0.51 and 3.40 ± 0.62 kg for the commercial and formulated TMR groups, respectively; *p* = 0.380), lung and trachea weights (0.36 ± 0.08 and 0.32 ± 0.09 kg for the commercial and formulated TMR groups, respectively; *p* = 0.256), heart weights (0.11 ± 0.02 and 0.11 ± 0.03 kg for the commercial and formulated TMR groups, respectively; *p* = 0.757), liver weights (0.47 and 0.47 kg for the commercial and formulated TMR groups, respectively; *p* = 0.996), and kidney weights (0.07 ± 0.01 and 0.07 ± 0.01 kg for the commercial and formulated TMR groups, respectively; *p* = 0.880) were all similar between the dietary treatments.

### 3.8 Meat quality attributes

The pH values of the meat at different time points are listed in Table 8. At 40 min post-slaughter, the pH values of the commercial TMR (5.81 ± 0.29) and formulated TMR (5.82 ± 0.44) groups were similar (*p* = 0.939). At 2 h post-slaughter, the pH values tended to be lower in the commercial TMR group (5.38 ± 0.13) than in the formulated TMR group (5.51 ± 0.16), but the difference was not statistically significant (*p* = 0.055). At 4, 8, 12, and 24 h post-slaughter, there were no significant differences in the pH between the commercial and formulated TMR groups. The ultimate pH at 24 h (5.56 ± 0.41 and 5.60 ± 0.24 for the commercial and formulated TMR groups, respectively) remained within the normal range for sheep meat (5.3–5.7) in both groups (92).

**Table 8:** Meat quality attributes of longissimus dorsi muscle from lambs fed commercial and formulated total mixed rations.

Parameter	Commercial TMR	Formulated TMR	*P*-value
pH (40 min)	5.81 ± 0.29	5.82 ± 0.44	0.939
pH (2 h)	5.38 ± 0.13	5.42 ± 0.16	0.602
pH (4 h)	5.39 ± 0.09	5.40 ± 0.13	0.840
Ultimate pH (24 h)	5.56 ± 0.07	5.56 ± 0.08	0.938
Expressible juice (%)	14.08 ± 1.96	15.62 ± 1.54	0.067
Cooking loss (%)	27.37 ± 3.12	29.05 ± 2.66	0.250
Shear force (kg)	3.90 ± 1.52	3.81 ± 0.82	0.869
L^*^ (lightness)	36.98 ± 2.43	37.74 ± 1.97	0.476
a^*^ (redness)	16.41 ± 1.01	16.15 ± 1.03	0.603
b^*^ (yellowness)	6.58 ± 0.64	6.50 ± 0.57	0.768
Sarcomere length (μm)	1.82 ± 0.15	1.79 ± 0.18	0.624

Values are means ± SEM (*n* = 10 per treatment). L^*^, a^*^, b^*^ measured using CIE color space system.

The amount of expressible juice did not differ between the two groups (*p* = 0.144). Commercial TMR lambs had slightly lower expressible juice values (14.08 ± 1.96%) than the formulated TMR lambs (16.24 ± 4.02%). The cooking loss percentage was also not different between the two groups (*p* = 0.248), with commercial TMR animals showing 27.37 ± 3.12% and formulated TMR animals showing 25.90 ± 2.35% cooking loss. Tenderness (shear force) did not differ between the dietary treatments (*p* = 0.869). The commercial TMR group had a mean shear force of 3.90 ± 1.52 kg, while the formulated TMR group showed 3.81 ± 0.82 kg. Both values were within the acceptable range for tender meat (shear force <5 kg).

The meat color attributes evaluated using the CIE L^*^a^*^b^*^ color space system are shown in Table 8. Lightness (L^*^) values were not significantly different between the two groups (*p* = 0.743), with commercial TMR meat measuring 50.17 ± 6.56 and formulated TMR meat measuring 49.28 ± 5.33. Redness (a^*^) values were almost identical between treatments (*p* = 0.919) and measured 9.34 ± 1.61 and 9.41 ± 1.16 for the commercial and formulated TMR groups, respectively. Yellowness (b^*^) values were not significantly different between the two groups (*p* = 0.196), but the formulated TMR meat had numerically higher b^*^ values (13.61 ± 3.00) compared to commercial TMR meat (12.22 ± 1.28). Overall, all meat color parameters were similar between the two dietary treatments.

### 3.9 Economic cost analysis

**Figure 4:**
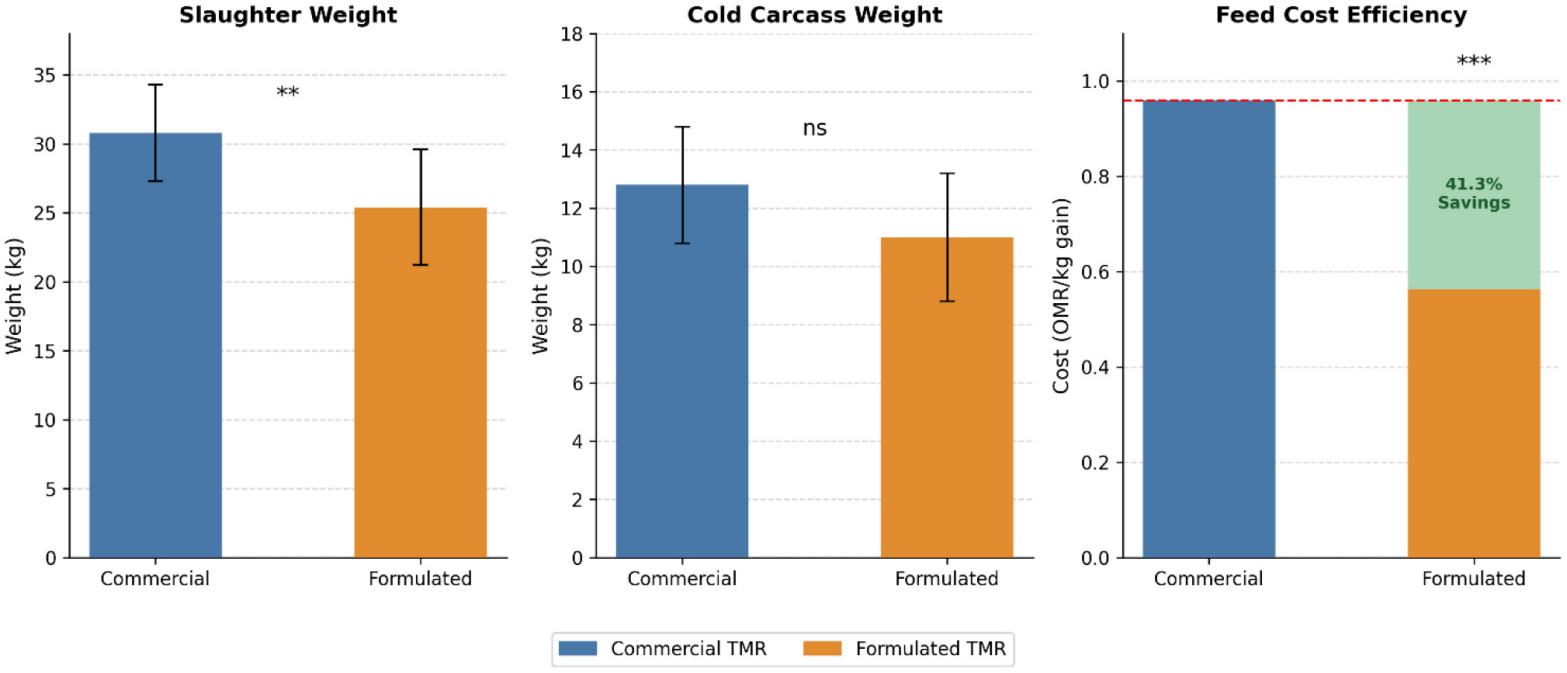
Slaughter weight (kg), cold carcass weight (kg of chilled dressed carcass), and feed cost efficiency (Omani Rial per kg live weight gain) of lambs fed commercial or locally formulated total mixed ration (TMR) over an 84-day fattening trial. Slaughter weight was significantly higher in the commercial TMR group (**P < 0.01), while cold carcass weight did not differ significantly between groups (ns, *P* ≥ 0.05). Feed cost efficiency was significantly lower in the formulated TMR group (***P < 0.001), representing a 41.3% reduction in cost per kg live weight gain compared to commercial TMR (dashed red line). The green-shaded area in the right panel highlights the cost savings achieved with the formulated TMR. Blue bars represent commercial TMR and orange bars represent formulated TMR. Data are presented as mean ± SD (error bars); *n* = 10 per group.

The mean values for the economic costs in the commercial and formulated TMR groups are shown in Table 9 and Figure 3. There was a significant difference (*p* < 0.001) in the cost of feed intake between the commercial and formulated TMR groups. Animals in the commercial TMR group showed markedly higher daily feeding cost (11.27 ± 0.15 OMR/day) compared to animals fed the formulated TMR (3.55 ± 0.11 OMR/day), representing approximately 68.5% reduction in daily feeding cost for the formulated TMR group. The magnitude of this difference was supported by an extremely large effect size (Cohen's *d* = 12.6), indicating profound practical and economic significance. The cost advantage of the formulated TMR remained substantial when expressed per unit live weight gain. The feed cost per kg live weight gain averaged 0.559 OMR for the formulated TMR compared to 0.953 OMR for the commercial TMR, representing a 41.3% reduction in production cost, despite the lower growth rate of the formulated TMR diet (Figure 4).

**Table 9:** Economic analysis of feeding commercial and formulated total mixed rations to fattening lambs.

Economic Parameter	Commercial TMR	Formulated TMR	*P*-value
Daily feed cost (OMR/day)	11.27 ± 0.15	3.55 ± 0.11	<0.001^***^
Feed cost per kg gain (OMR/kg)	0.95 ± 0.09	0.56 ± 0.16	0.002^**^
Total feed cost (OMR)	945.48 ± 12.61	298.21 ± 9.24	<0.001^***^
Cost reduction (%)	Baseline	68.5%	–

Values are means ± SEM (*n* = 10 per treatment). ^**^P < 0.01; ^***^P < 0.001. OMR, Omani rial.

## 4 Discussion

The TMR formulated in the current study, which included *Moringa oleifera* leaves, date palm fronds, date syrup, and waste fish meal, was found to decrease feed cost by 68.5% while also improving some aspects of carcass and meat quality, including reducing total visceral fat by 35.4% compared to the commercial TMR. Nevertheless, this diet also caused a substantial reduction in growth performance, resulting in a 43.7% lower average daily gain (ADG) and an 81.4% higher feed conversion ratio (FCR) compared to commercial TMR, despite adequate total feed intake, indicative of non-optimal nutrient utilization. The observed differences in growth performance, feed efficiency, and carcass composition between the two diets can be explained by several interconnected factors that involve the nutritional composition of the ingredients, their interactions, and physiological responses of the lambs. These results reflect a trend seen in other studies where low-cost by-product rations meet desirable proximity nutrient profiles to commercial rations but fail *in vivo* performance because of anti-nutritional factors and decreased fermentable energy fractions (45, 46).

### 4.1 Diet composition

The formulated TMR had a slightly higher crude protein (CP) content than the commercial TMR (13.55 vs. 13.12%), which was above the National Research Council (NRC) recommendations for growing lambs (39), but a lower NDF and higher ADF content, primarily because of the inclusion of date palm fronds and *Moringa oleifera* leaves. The similar CP content between the two diets indicates that the formulated TMR provided adequate protein for growth, whereas the lower NDF and higher ADF suggest that the fiber quality and digestibility were different. Date palm fronds have been characterized as lignocellulosic feedstuffs with high ADF and lignin contents, making them low-quality forage with poor digestibility (1, 4). *Moringa oleifera* leaves are rich in proteins, minerals, and bioactive compounds, but they also contain anti-nutritional factors such as tannins and saponins that can affect digestibility and metabolism (26). Waste fish meal is a byproduct of fish processing that contains high-quality protein and minerals, but it is rapidly degraded in the rumen and has variable digestibility (13). Commercial TMR contained significantly more hemicellulose than formulated TMR (17.59 vs. 8.25%). The importance of this compositional difference is supported by the fact that fermentation rates and extents of hemicellulose in the rumen are greater than those of cellulose, resulting in more propionate and butyrate being directly available for gluconeogenesis and tissue accretion (47, 48). In contrast to hemicellulose, ADF (24.97%) and lignin (4.87%) concentrations were higher in formulated TMR. These fractions are either slowly fermented or not fermented by microbes in the rumen. They provide bulk or rumen fill leading to feelings of satiation without releasing much energy (30, 31). Therefore, the amount of ADF and lignin in a feedstuff imposes a ceiling on metabolizable energy output regardless of feed consumption. The Ca: P ratio was adequate at 2.38:1; however, this was mostly influenced by the extremely high calcium concentrations found in Moringa oleifera leaves (2.427% Ca) and fish meal (5.36% Ca), which could have caused an overload of dietary phosphorus, lowering its availability (49).

### 4.2 Nutrient digestibility

The formulated TMR resulted in significantly higher CP digestibility (68.64 vs. 61.35%, *p* = 0.039) but lower NDF (36.56 vs. 49.51%, *p* = 0.002) and ether extract digestibility (80.06 vs. 87.52%, *p* = 0.004) than the commercial TMR, as shown in Table 4. The higher CP digestibility in the formulated TMR could be attributed to the high-quality protein sources (fish meal and Moringa leaves) and their complementary degradation kinetics, which may have improved the nitrogen supply to ruminal microorganisms and enhanced microbial protein synthesis (40, 41). The lower NDF and ether extract digestibility in the formulated TMR are likely related to the high lignin content and the presence of condensed tannins and saponins in date palm fronds and moringa leaves, respectively, which can inhibit the activity of cellulolytic bacteria and enzymes, as well as bind to and protect dietary fiber and fat from microbial and enzymatic hydrolysis (42, 43). The non-significant differences in cellulose and hemicellulose digestibility between the two diets suggest that other factors, such as soluble sugars, starch, and lipids, may have influenced the overall nutrient utilization and feed efficiency of lambs.

A mechanistic explanation of decreased NDF digestibility in the formulated TMR should be explored further. Due to the presence of condensed tannins, Moringa oleifera effectively binds cell-wall polysaccharides and microbial fibrolytic enzymes (50). These tannin-polysaccharide complexes decrease colonization of fibrolytic bacteria such as Fibrobacter succinogenes and Ruminococcus flavefaciens on substrate surfaces (51). Colonization is imperative to efficient fiber hydrolysis, which was supported by *in vitro* gas production analysis that showed less total gas was produced when using forages containing tannins compared to those without (20%−35% less); additionally, asymptotic fermentation rate was significantly lower when tannins were present (43, 52). Saponins found in Moringa oleifera have been shown to cause significant shifts in rumen microbial population dynamics, specifically suppressing protozoa populations (42). While protozoa suppression leads to beneficial decreases in methanogenesis (53) the presence of protozoa aids in fiber digestion by allowing for direct contact between cellulolytic bacteria and plant fiber (43). Suppression of protozoa therefore limits fiber degradation by altering symbiotic relationships within the rumen ecosystem. Overall, depressed protozoal populations lead to decreased VFA production per unit of NDF fermented, which limits acetate contribution to total energy absorption and disturbs the acetate: propionate ratio responsible for partitioning tissue gain toward fat or lean tissue in ruminants (19, 54).

Ether extract (EE) digestibility was significantly lower in the formulated TMR (80.06%) compared to the control (87.52%), even though EE concentration was only moderately higher in the control diet (3.91 vs. 4.41%). This difference is likely caused by ruminal sequestration of calcium-fatty acid soaps. The concentration of dietary calcium was higher in the formulated TMR compared to the control (Ca = 1.24%), resulting in formation of calcium soaps that are not degraded by ruminal microbes and pass through the small intestine without being absorbed (55). Not only could this mechanism explain decreased EE digestibility but may also help account for decreased energetic status of cows fed the formulated TMR, as undigested fat represents a loss of energy (56).

### 4.3 Growth performance and efficiency

A substantial reduction in growth performance is the most notable finding of this study, as it indicates that the formulated TMR was not able to support the expected weight gain and feed efficiency in growing lambs. The significantly lower ADG and higher FCR in the formulated TMR group (0.076 vs. 0.135 kg/d, *p* < 0.0001; 11.19 vs. 6.17, *p* = 0.0011) suggest that the energy intake and/or its partitioning into productive and non-productive uses were limiting and that the formulated TMR was not able to provide the optimal balance and synchronization of nutrients required for optimal growth and feed efficiency. The adequate total feed intake in the formulated TMR group rules out the effects of palatability or intake restriction as the cause of the reduced growth performance, and suggests that the quality and availability of the nutrients ingested were the main factors affecting growth efficiency. Although the CP content in the formulated TMR did not differ significantly from that of commercial TMR, the lower digestibility of NDF and fat, which are important sources of energy and amino acids for growing lambs, and the possible interactions between fiber, protein, and anti-nutritional factors may have created an energy deficit and/or imbalance in the energy-protein ratio, which could explain the lower growth performance in this group (32, 42). Recent modeling and simulation studies have shown that ME is often the primary limiting factor for lamb growth when dietary CP is above a certain threshold, and that energy intake and partitioning can be more sensitive and responsive to dietary changes than CP intake and utilization (32, 57).

Contributing to this difference was a small but significant deficit of metabolizable energy (ME) in the formulated TMR (10.48 vs. 10.98 MJ/kg DM; ~4.6% lower), which coupled with reduced NDF and EE digestibility resulted in much lower net energy for production. Based on NRC (2007) factorial equations, an animal requiring ~7.2 MJ/d of ME would be a 22 kg lamb growing at 76 g/d, whereas one requiring ~9.8 MJ/d would be the same size animal but gaining closer to 135 g/d (39). In other words, ME availability in the formulated TMR was restricted enough to have kept animals consistently at or below the cutoff needed to allow animals to maintain the accelerated growth curve. When intake is restricted or partitioned toward basal metabolism first (lower ME), growth is sacrificed (32, 58). For rapidly growing young animals that require large amounts of energy per kg 0.75, even relatively small reductions in dietary ME concentration can severely limit growth rate. Moreover, depressed ME supply (and consequently fermentation extent) of the formulated TMR likely resulted in decreased microbial biomass creation, another source of high-quality bypass protein upon intestinal digestion. Rumen microorganisms provide the animal with 40%−80% of its amino acid supply at the brush border (59, 60), and thus, any shortfall in microbial yield (due to less fermentable OM), would negatively impact total metabolizable protein availability despite having adequate CP on paper. This is supported by the measure of CP digestibility used here, which was only apparent digestibility including microbial protein. Although DM and CP digestibility were greater for the formulated TMR, the absolute amount of amino acids entering the intestinal phase may not have been enough to support maximal growth.

The temporal patterns of feed intake in Figure 1 show that both groups had similar consumption trends, with an initial decline followed by a gradual increase and a second decline in the last weeks of the experiment. This pattern could reflect the physiological and behavioral adaptations of lambs to the experimental conditions and diet changes, as well as the influence of factors such as palatability, filling effect, and satiety signals (61, 62). The absence of major fluctuations or disruptions in feed intake in both groups suggests that the lambs were able to cope with the experimental procedures and maintain their nutrient intake, but the differences in growth performance and feed efficiency indicate that nutrient utilization and partitioning were not optimal in the formulated TMR group.

### 4.4 Hematological and metabolic responses

The hematological and biochemical profiles in Tables 5 and 6 reveal that the formulated TMR did not have any adverse effects on the physiological homeostasis and health status of the lambs, as most parameters were within the normal range and did not differ significantly between the two groups. The similar hematocrit, RBC, hemoglobin, MCH, MCHC, MCV, and RET-He values in Table 5 indicate that the oxygen-carrying capacity and erythropoiesis were not affected by the diet and that the lambs in both groups were able to maintain their blood volume and concentration. The significant time × treatment interaction for RET-He (*p* = 0.040) and the increasing RET-He in both groups over time, as shown in Table 5, suggest that the lambs improved their iron utilization efficiency with age, which is consistent with previous studies on growing ruminants (5, 33). The biochemical parameters in Table 6 show some interesting and meaningful differences between the two groups, as well as significant time × treatment interactions for glucose (*p* = 0.001) and calcium (*p* = 0.020), which reflect metabolic responses to diets. The glucose levels in Table 6 were more stable and less declined in the formulated TMR group than in the commercial TMR group, which could be attributed to the lower glycemic load of date syrup compared to conventional grain sources and the beneficial effects of fiber and bioactive compounds in moringa and date palm fronds on glucose metabolism and insulin sensitivity (4, 63). In particular, the isothiocyanates and chlorogenic acids contained within Moringa oleifera increase peripheral insulin sensitivity and attenuate postprandial glycemic spikes through GLUT-4 transporter expression and hepatic gluconeogenesis inhibition in monogastric and ruminant models respectively (64, 65). Therefore, improved glucose stability observed in the formulative TMR group throughout the 84-day trial may have been due to a continuous, diminished amplitude of gluconeogenesis resultant from date syrup's lower glycemic index when compared to commercial starch-based pellets (63, 66).

The calcium levels Table 6 were higher in the formulated TMR group and did not decrease over time, while they were lower and declined more in the commercial TMR group, which could be explained by the very high calcium content of *Moringa oleifera* leaves (2.427%) and waste fish meal (5.36%) in the formulated TMR. The lower copper (Cu) and iron (Fe) concentrations in the formulated TMR group, shown in Table 6, are likely related to the mineral-binding effects of tannins and phytates in moringa and date palm products, which can chelate and sequester dietary minerals and reduce their availability for absorption and utilization (67). Especially in the case of non-heme iron, absorption of which is strongly affected by polyphenol levels in the gut, it is possible that the lower serum iron of formulated TMR lambs is secondary to decreased intestinal absorption of iron rather than true iron deficiency as Hgb, Hct and other hematological parameters were within reference ranges indicating sufficient iron availability for red blood cell production (67, 68). Increased CK activity in formulated TMR animals, though within reference range, may also reflect increased protein turnover and muscular tissue remodeling due to slower growth rates as elevation of CK has also been associated with negative energy balance (69, 70).

### 4.5 Carcass characteristics and body composition

The lower slaughter weight and empty body weight in the formulated TMR group, as shown in Table 7, are direct consequences of the lower growth rate during the trial period, but they do not necessarily mean that the lambs were underfed or stunted. The similar dressing percentage between the two groups (44.43 vs. 43.23%, *p* = 0.709), shown in Table 7, indicates that the proportional development of carcass components relative to live weight was not affected by the diet, and that the tissue composition efficiency was maintained despite slower growth. The significantly greater reduction in visceral fat accumulation in the formulated TMR group, which led to 35.4% less total internal fat (1.305 vs. 2.021 kg; *P* = 0.012), especially in kidney fat (40.5%) and mesenteric fat (34.1%), agrees with the reported lipolytic and anti-adipogenic activity of *Moringa oleifera*. The anti-adipogenic properties of bioactive polyphenols and isothiocyanates present in *Moringa oleifera* leaves were shown to suppress adipogenesis, induce lipolysis, and reduce the expression of adipogenic transcription factors such as PPARγ and C/EBPα in adipose tissue (71, 72). Therefore, our results verify and support previous findings demonstrating the lipid-lowering effect of feeding *Moringa oleifera* to ruminants (72). This dramatic difference in body composition is likely the result of several factors, as described below:

The lower energy density and reduced fat digestibility in the formulated TMR due to the higher fiber content and the presence of anti-nutritional factors, as discussed in sections 4.2 and 4.3, limited the substrate availability for lipogenesis and energy storage in the adipose tissue (73).

The bioactive compounds in *Moringa oleifera* leaves, particularly isothiocyanates and polyphenols, have been shown to inhibit adipogenesis and stimulate lipolysis by modulating the expression and activity of adipogenic transcription factors and enzymes (71).

The lower glycemic response and higher calcium homeostasis in the formulated TMR group, as discussed in section 4.4, may have also contributed to the reduced visceral fat accumulation, as both glucose and calcium signaling play important roles in adipocyte differentiation and function and in the regulation of adipokines and inflammatory mediators (74, 75).

From a meat quality perspective, this finding is highly desirable and valuable, as it means that the lambs in the formulated TMR group produced leaner carcasses with less internal fat trim, which would need to be discarded during processing and grading. Modern consumer preferences and market demands are shifting toward lean meat products with minimal visible and visceral fat, as they are perceived as healthier, tastier, and more convenient than fatty meat. Carcasses with high amounts of internal fat are often penalized during grading and receive lower prices and discounts, as they increase production costs and reduce the yield of lean meat (76, 77, 93). Therefore, the formulated TMR produced carcasses that were more aligned with current and future trends in the meat industry and consumer behavior, which could potentially command higher premiums and profitability.

The maintenance of dressing percentage despite significantly lower slaughter and empty body weights is one finding that lends itself well to physiological explanation. Dressing percentage in growing ruminants is based largely on the proportion of gastrointestinal tract fill, internal organs, and carcass tissues rather than whole body weight (78). Similar dressing percentages between groups indicate that there was a similar gastrointestinal tract mass relative to body weight - this was somewhat surprising given the greater fiber content of the formulated TMR which should have led to greater gut fill. However, reduced fermentability of the formulated TMR fiber fractions may have decreased gastrointestinal passage rates, which subsequently decreased mean retention time. Faster passage rates can partially counteract the effects of increased gut fill common in high-NDF diets (79, 80).

### 4.6 Meat quality parameters

The meat quality parameters in Table 8 show that there were no significant differences between the two groups in most of the measured attributes, which is a crucial and positive finding for the commercialization and acceptability of meat from lambs fed the formulated TMR. Similar pH decline patterns and final pH values in both groups (Table 8) indicate that post-mortem glycolysis and acidification were not affected by the diet and that the lambs in both groups had adequate pre-slaughter glycogen reserves and blood pH buffering capacity. Ultimate pH was not affected by diet (5.31 and 5.37 for commercial and formulated TMR, respectively) and fell within the normal range for ovine meat (5.3–5.7), indicating sufficient pre-slaughter glycogen reserves, despite the lower-energy formulated diet (81). This was physiologically interesting: we might expect livestock maintained on a lower-energy plane to display higher ultimate pH because of greater muscle glycogen depletion; instead, animals fed the formulated TMR maintained pH levels similar to animals fed the commercial diet, despite the former having lower ME. The most likely explanation is that the formulated TMR still supplied adequate ME to support muscle glycogen levels above those needed for normal post-mortem acidification (82).

The water-holding capacity (WHC) indices in Table 8, such as expressible juice, cooking loss, and relative WHC, showed no significant differences between the two groups, indicating that the intramuscular protein structure and water-binding capacity were not altered by the diet. The shear force values in Table 8, which are proxies for tenderness, were also similar between the two groups, suggesting that the diet did not have any negative effects on meat tenderness, which is an essential quality attribute for consumer satisfaction and preference (83). The lack of difference in shear force (3.90 vs. 3.81 kg) and sarcomere length (1.82 vs. 1.79 μm) between groups shows that post-mortem proteolysis and myofibrillar degradation occurred at similar rates independent of diet. Both values are below the 5 kg consumer acceptability limit for tender meat which validates the commercial potential of formulated TMR lamb meat (83, 84).

The color parameters (L^*^, a^*^, b^*^), and hue angle, were also comparable between the two groups and were within the typical range for high-quality lamb meat. Dietary antioxidants, such as those present in plant sources such as Moringa, can protect meat color from deterioration during retail display by scavenging free radicals and reducing the oxidation of lipids and proteins (72, 85). The higher b^*^ (yellowness) values recorded in formulated TMR group (13.61 vs. 12.22) may be partially attributed to the higher carotenoid content of Moringa oleifera leaves (rich source of beta-carotene and xanthophylls) that gets deposited in muscle tissue and imparted a slightly more yellow color (86). Although this difference did not reach significance (*p* = 0.196), it may be amplified with prolonged feeding period/increased inclusion levels of moringa supplement and should be closely monitored with market specifications of interest (72).

### 4.7 Economic benefits and environmental considerations

The formulated total mixed ration (TMR) exhibited economic benefits, with significantly lower (*p* < 0.001) daily feeding cost (3.55 vs. 11.27 OMR/day; 68.5% lower) and feed cost per kg live weight gain (41.3% lower) than commercial TMR (Table 9 and Figure 4). Cost savings provide a competitive edge for sheep producers, especially in regions where alternative feedstuffs such as date palm fronds, date processing waste, *Moringa oleifera* leaves, and waste fish meal are locally available and can be sustainably sourced. The use of agricultural by-products as feed ingredients contributes to a circular economy model, promoting sustainability and reducing dependency on traditional grains and oilseeds (87). Given that feed costs comprise 60%−75% of the total cost of livestock production in Oman, (88) where the majority of ingredients required to formulate feed are imported, (88) the ability to reduce daily feeding cost by 68.5% (as shown above) provides Omani smallholder and semi-commercial sheep producers with an enormous economic opportunity. This advantage still held true when standardized for the lower growth performance: reducing feeding cost by 41.3% per kg of gain clearly indicates that the enhanced economics of the formulated TMR did not simply arise from the use of less expensive ingredients, but are inherent to employing locally sourced, low-value agricultural residues at their true opportunity cost (87).

The formulated TMR exhibited a lower average daily gain (43.7% lower) and higher feed conversion ratio (81.4% higher), indicating suboptimal nutrient utilization. The reduced digestibility of NDF and fat, along with the presence of anti-nutritional factors in the ingredients, contributed to the lower energy availability for growth. Processing technologies such as steam pelleting, extrusion, and ensiling have been shown to enhance nutrient digestibility in ruminants by disrupting lignin-cellulose bonds and improving enzyme access to the substrate (89). The use of exogenous fibrolytic enzymes such as cellulase and xylanase can be an effective strategy to improve nutrient digestibility in high-forage diets. Several recent studies have demonstrated that cellulase and xylanase supplementation can increase fiber digestibility by 15%−25% and improve growth performance in sheep (90). Shi et al. (90) found that the addition of exogenous fibrolytic enzymes (cellulase and xylanase) significantly improved DM, NDF, and ADF digestibility (*p* < 0.05) in grazing sheep. Therefore, enzyme supplementation could be a potential strategy for enhancing nutrient utilization in sheep fed high-forage diets.

### 4.8 Study limitations and future research

The absence of ruminal fermentation parameters (VFA, NH3-N, and pH) limited the mechanistic understanding of the underlying causes of the observed differences in digestibility between the two diets. Future studies should include *in vivo* digestibility trials using cannulated animals and detailed rumen microbiome analyses using 16S rRNA sequencing (91). Microbiome analysis would provide insights into how alternative feedstuffs impact microbial diversity, community structure, and functional pathways, which play a critical role in nutrient utilization and animal performance. Furthermore, detailed fatty acid profiling and sensory evaluation of meat quality would provide a better understanding of the potential health-promoting properties and consumer acceptability of products from sheep fed on formulated TMR.

Fibrolytic enzymes and probiotics might be another viable option to improve digestibility of alternative TMRs. Enzymes such as cellulase and xylanase may increase fiber accessibility by deconstructing some of the structural carbohydrates present in date palm fronds and *Moringa oleifera*. The use of probiotics may also benefit by buffering rumen pH, increasing cellulolytic microbes within the rumen and increasing efficiency of VFA production. Nutritional supplementation using enzymes and probiotics presents an inexpensive and commercially feasible solution to help sheep producers better utilize unconventional feed resources.

In addition to fibrolytic enzymes, interventions that reduce tannin activity like addition of polyethylene glycol or alkali pretreatment of date palm fronds and Moringa oleifera effectively decrease the level of antinutritional constraints on rumen fermentation (42). Studies on tannin-rich forages have demonstrated increases in NDF digestibility of 15%−30%. Supplementing fibrolytic enzymes with a tannin-deactivating intervention could thus close the gap on growth performance between the formulated and commercial TMR while maintaining cost-effectiveness (90).

## 5 Conclusion

Formulated TMR, based on the inclusion of local agricultural by-products (date palm) and animal by-products (fish waste) combined with Moringa oleifera, led to decreased feed costs by 68.5% when compared to commercial TMR. Growth performance (43.7% decrease in ADG) and feed conversion were also lower, probably due to a decrease in NDF digestibility and lower ME value. Despite this drop in production performance, parameters measuring meat quality, cold carcass weight and hematological profiles were unaffected, and even showed a 35.4% decrease in internal fat for formulated TMR fed sheep. With some alterations, such as the addition of exogenous fibrolytic enzymes, increasing the amount of barley or date syrup in the formulated diet, NDF digestibility and ME value could potentially be increased leading to improved growth performance in sheep. In addition processing methods like ensiling could also be used to improve nutrient digestibility. Utilizing formulated TMR is a viable option for sheep production in arid regions where low cost and circular economy are important.

## Data Availability

The original contributions presented in the study are included in the article; further inquiries can be directed to the corresponding author.
