# Correction: Date palm by-products, fish waste, and *Moringa oleifera* as a cost-effective total mixed ration for fattening lambs: trade-offs between economic benefits and growth performance

**DOI:** 10.3389/fvets.2026.1893441

**Published:** 2026-06-11

**Authors:** Said Al-Khalasi, Abdullah Al-Ghafri, Fahad Al-Yahyaey, Kaadhia Al-Kharousi, Suad Al-Saqri, Zainab Al-Ismaili, Hala Al-Sheibani

**Affiliations:** 1 UNESCO Chair on Aflaj Studies and Socio-hydrology, University of Nizwa, Nizwa, Oman; 2 Animal Production Research Center, Ministry of Agricultural, Fisheries Wealth and Water Resources, Barka, Oman; 3 Animal and Veterinary Sciences, Sultan Qaboos University, Muscat, Oman; 4 Department of Biological Sciences and Chemistry, University of Nizwa, Nizwa, Oman

**Keywords:** alternative feedstuffs, carcass characteristics, date palm by-products, feed digestibility, lamb fattening, *Moringa oleifera*, total mixed ration, economic analysis

There was a mistake in the caption of **Figure 3**, as published.

The incorrect caption stated:

“Slaughter weight, cold carcass weight (weight of chilled dressed carcass), and feed cost (Omani Rial per kg live weight gain) as affected by feeding commercial vs. locally formulated total mixed ration (TMR). Even though slaughter weight was significantly higher in commercial group (^**^P < 0.01), cold carcass weight was unaffected (ns, *P* ≥ 0.05) and feed cost was reduced by 41.3% with formulated TMR feed (^**^P < 0.001). Dashed red line in right panel represents cost of commercial group. Data are presented as mean ± SD (error bars).”

The corrected caption of **Figure 3** appears below.

**Figure 3**. Bar chart comparing apparent digestibility percentages of dry matter, organic matter, crude protein, NDF, ADF, and ether extract between lambs fed commercial TMR (blue bars) and locally formulated TMR (orange bars). Significant differences indicated for crude protein (^*^P < 0.05) and NDF (^**^P < 0.01). Error bars = SEM; *n* = 4 per group”.

**Figure 3** was placed in the wrong order in the PDF and HTML version of this paper. Following sequential order, **Figure 3** has now been renamed **Figure 1**. All figures have been renumbered, and their order has been corrected.

The original version of this article has been updated.

